# Sol-Gel Multilayered Niobium (Vanadium)-Doped TiO_2_ for CO Sensing and Photocatalytic Degradation of Methylene Blue

**DOI:** 10.3390/ma17081923

**Published:** 2024-04-22

**Authors:** Simeon Simeonov, Anna Szekeres, Maria Covei, Hermine Stroescu, Madalina Nicolescu, Paul Chesler, Cristian Hornoiu, Mariuca Gartner

**Affiliations:** 1 Institute of Solid State Physics, 72, Tsarigradsko Chaussee, 1784 Sofia, Bulgaria; simeon@issp.bas.bg (S.S.); szekeres@issp.bas.bg (A.S.); 2 Department of Product Design, Mechatronics and Environment, Transilvania University of Brasov, 29 Eroilor Bd., 500036 Brasov, Romania; maria.covei@unitbv.ro; 3 Institute of Physical Chemistry “Ilie Murgulescu”, Romanian Academy, 202 Splaiul Independentei, 060021 Bucharest, Romaniachornoiu@icf.ro (C.H.)

**Keywords:** titanium dioxide, Nb(V) doping, sol-gel TiO_2_ structure, electrical properties, CO sensing, photocatalytic methylene blue degradation

## Abstract

Multilayered TiO_2_ films doped either with Niobium or Vanadium (1.2 at. %) were deposited by the sol-gel dip coating method on c-Si and glass substrates. The films on glass substrates were tested for CO sensing and photocatalytic degradation of methylene blue. X-ray diffraction data analysis showed that all the TiO_2_:Nb(V) films were nanocrystalline in the anatase phase, with a uniform and compact microstructure and a homogeneous superficial structure of small grains with diameters in the range of 13–19 nm. For the electrical characterization, the TiO_2_:Nb(V) films were incorporated in Metal-Insulator-Semiconductor (MIS) structures. The specific resistivity is of the order of 10^4^ Ωcm and its value decreases with increasing the electrical field, which testifies to the injection of electrons into these layers. From the analysis of the current–voltage curves taken at different temperature- and frequency—dependent capacitance–voltage and conductance–voltage characteristics, the density and parameters of deep levels in these TiO_2_ films are evaluated and the electron charge transport mechanism is established. It was shown that the current in these TiO_2_:Nb(V)-Si MIS structures is mainly carried out by inter-trap tunneling via deep levels energetically distributed in the TiO_2_ bandgap. Testing these sol-gel TiO_2_:Nb(V) layers for gas sensing and photocatalytic capabilities proved that they could serve such purposes. In particular, the results of the V-doped sol-gel TiO_2_ film confirm its CO detection capability, which is rarely reported in the literature. For the photodegradation of methylene blue, the Nb-doped TiO_2_ samples were superior, with nearly double the photocatalytic efficiency of undoped TiO_2_.

## 1. Introduction

Titania (TiO_2_) possesses unique properties that put it among the most frequently studied materials. Thin TiO_2_ films probably have the broadest application areas in electronic devices, as well as in both the metallurgical and food industries or medicine. For these applications, mainly the anatase [1,2,3] and rutile [4,5] TiO_2_ crystalline phases are of interest and are extensively studied. TiO_2_ has a high dielectric permittivity and refractive index, and good transparency in the visible spectral range of light, as well as high chemical stability and photocatalytic activity [6].

Stoichiometric TiO_2_ is an insulator at room and moderate temperatures with considerably wide energy bandgap values (*E_g_* > 3.0 eV) and has an extremely high specific resistivity of ρ ≥ 10^8^ Ωcm [7]. This is a serious impediment for many industrial applications of TiO_2_ coatings. This problem could be solved by fine-tuning the *E_g_* value by introducing intrinsic or extrinsic defects (dopants) that create localized states in the TiO_2_ energy bandgap, thus changing the properties of the films [8,9].

TiO_2_ thin films, deposited by different techniques, are non-stoichiometric semiconductors and their properties are mainly determined by the O/Ti ratio and the defect disorder in the deposited films [10]. In an oxygen-deficient TiO_2_ film, the predominant defects are oxygen vacancies and Ti interstitials [11]. These defects are donor-like and that is why the undoped TiO_2_ is an n-type semiconductor. On the contrary, when the films are grown in an oxygen-rich environment, the predominant defects are Ti vacancies, which are acceptor-type defects transforming the films into a p-type semiconductor [12]. The introduction of metallic or non-metallic dopants into the growing TiO_2_ films modifies not only the electronic band structure of TiO_2_ but also the structural, optical and electrical properties of the formed layers [6,13,14].

The preparation method of TiO_2_ films is a decisive factor determining the film’s structure. Predominately, the deposited TiO_2_ coatings have a nanocrystalline (nc) structure and the phase and size of the crystallites are highly dependent on the applied technological conditions. There are several physical and chemical deposition techniques with their own advantages and disadvantages [15,16]. Physical methods require expensive equipment and a complex process running at higher temperatures, while chemical methods are more accessible and cheaper. It has been shown that for applications where a large surface area is required, such as gas sensing and photocatalytic processes, reducing the particle size improves various working parameters of the TiO_2_ films [8,17,18], highlighting the superior advantage of the nanocrystalline structure. For example, CO, NO_2_ and hydrocarbons, very harmful atmospheric pollutants resulting from automobile exhaust and industrial processing, can be detected using TiO_2_-based chemosensors [19,20]. TiO_2_ is thermally stable but its high electrical resistance is a drawback when used in applications such as sensor devices in the automotive exhaust [21]. Commercial sensors used in practical applications often require very high operating temperatures (1000 °C), high thermal stability, and long life-operation. Doping can be used to improve the gas-sensing characteristics of TiO_2_ films (lower sensor operating temperatures, and environmentally friendly fabrication techniques for the sensitive films).

From the above, it follows that by establishing the proper technology and finding suitable additives, the structure and properties of TiO_2_ thin films can be engineered in a controlled way according to the requirements of specific applications.

Our current scientific interest was focused on the study of TiO_2_ thin films. We chose the chemical sol-gel dip coating technique to deposit multilayered films layer-by-layer on Si and glass substrates. This chemical method is simple and allows for the precise control of the concentration of the dopant atoms. Our research has been motivated by a possible application of the sol-gel TiO_2_ films for sensor and/or photocatalytic applications. Two dopants, namely, Nb and V, were chosen, relying on reports related to their positive effect in these applications [8,22,23,24,25]. In order to reveal the influence of technological conditions on the structure and optical properties of these TiO_2_:Nb(V) films, extensive studies have been carried out [26,27,28]. We have established the optimal technological conditions which produce films with anatase phase and nano-scaled crystallites and good transparency as confirmed by XRD, TEM and transmittance measurements, respectively [27,28]. The AFM micrographs of the TiO_2_:Nb(V) multilayers have revealed uniformly distributed and quasi-spherically shaped nanoparticles, yielding smooth surfaces with a RMS roughness around 0.5 nm [28]. Preliminary electrical measurements of the obtained structures indicated the generation of defects states in the nc-TiO_2_ multilayers that mask the effect of dopant impurities [29,30]. In order to establish the origin and nature of these states, as well as their energy position in the TiO_2_ bandgap, a more thorough investigation of the electrical properties of these sol-gel nc-TiO_2_:Nb(V) multilayers is required.

In the present paper, we extended our earlier research on the changes in structural and electrical properties of TiO_2_:Nb(V) multilayers caused by the dopants. As an extension of the previously performed XRD studies, here, we elucidate the lattice strain generated by the sol-gel technology, and determine those parameters of the nanoparticles which play an important role in sensor and photocatalytic applications. Also, special attention is paid to establish the role of defect-generated deep levels in the charge carrier transport mechanism through TiO_2_:Nb(V) and to determine the parameters of these energy levels. The suitability of these TiO_2_:Nb(V) multilayers in gas sensing and photocatalytic applications is investigated by conducting a comparative study of the potential capability of the studied layers for these purposes. In this paper, the TiO_2_:Nb(V) films are studied for CO sensing under laboratory conditions (carrier gas was dry air), while their photocatalytic efficiency is tested in methylene blue (MB) degradation. The experimental results show that Nb(V) doping has little effect on the TiO_2_ structure but generates strains in the crystalline lattice and creates acceptor-type deep levels in the TiO_2_ energy gap as their charge compensates most of the shallow and deep donors in these films, keeping the resistivity at a considerably high value. The parameters of the deep levels in the TiO_2_ bandgap are evaluated and inter-trap tunneling as electron transport mechanism is revealed. The *I-V* characteristics of the TiO_2_:Nb(V)-Si heterostructures indicate a high rectification factor, which suggests promising areas for gas sensors and/or photocatalytic applications. The experiments conducted with these nc-TiO_2_:Nb(V) multilayers using them as gas sensor and photocatalyst support this statement. These gas-sensing results are novel and open a new perspective for a low-budget chemiresistor, based on V-doped TiO_2_ sensitive films, which can be used for CO detection.

## 2. Experimental Details

### 2.1. Material Preparation

We applied the sol-gel dip coating method to prepare TiO_2_ multilayers by successive deposition cycles of undoped and doped TiO_2_ with 1.2 at. % Nb or V atoms on substrates at room temperature. The preparation method is similar to that presented in [28]. Briefly, tetraethyl orthotitanate Ti(OC_2_H_5_)_4_ was used as a TiO_2_ source with the addition of acetylacetonate as a precursor to slow down the hydrolysis–polycondensation process to obtain thinner films. Niobium ethoxide [Nb(OCH_2_CH_3_)_5_] or vanadylacetylacetonate VO(C_5_H_7_O_2_)_2_ dissolved in ethanol (C_2_H_5_-OH) with 2,4-pentanedione as a chelating agent were used as the precursors of Nb and V dopants, respectively. The concentration of both metallic dopants was 1.2 at. %, following observations that to achieve higher doping efficiency in TiO_2_ and to avoid harmful lattice distortion, the concentration of doping metal ions should be considerably low [31,32]. The prepared sol-gel solutions were aged for 24 h before deposition procedures.

Before the sol-gel procedures, the Si substrates underwent standard RCA wafer cleaning, while the microscope glasses were cleaned in an ultrasonic ethanol bath and dried with a blow-drier. The substrates were immersed in the respective sol-gel solutions and withdrawn at a speed of 5 cm/min. After each deposition, the inter-layers were subjected to densification treatment at 300 °C for 30 min. After the deposition procedure was completed, the resulting TiO_2_ multilayers (5 and 10 layers in total) underwent a final heat treatment at 450 °C for 1 h. These films are further denoted as 5TiO_2_, 10TiO_2_ and 5TiO_2_:Nb(V), 10TiO_2_:Nb(V), respectively.

Depending on the measurement methods and objectives, sol-gel 5TiO_2_, 10TiO_2_ and 5TiO_2_:Nb(V), 10TiO_2_:Nb(V) films were deposited on p-type silicon as well as microscope glass substrates. Films on glass were used in the testing the films’ capability for gas sensing and as photocatalytic applications. 

For the electrical measurements, the deposited TiO_2_:Nb(V) layers were incorporated in Metal-Insulator-Semiconductor (MIS) structures formed by vacuum thermal evaporation of Al dots on the TiO_2_ top-surface through a metal mask and evaporation of a continuous Al layer on the silicon backside.

### 2.2. Measurement Methods

The thickness of the films and their optical constants were determined from spectroscopic ellipsometric (SE) spectra recorded in the UV/Vis/NIR spectral range by a J. A. Woollam Co. Inc. (Lincoln, NE, USA) variable-angle spectroscopic ellipsometer operating in the spectral range of (350–1700) nm and at variable angles of light incidence in the range of 45–90°. From the SE data analysis, we traced the change in the TiO_2_ bandgap’s width induced by the technological procedures and doping.

In the present paper, a more thorough investigation of the XRD patterns presented in [30,32] is made. These XRD spectra were recorded on an Ultima IV Diffractometer (Rigaku Corp., Tokyo, Japan) using CuKα radiation (*λ* = 0.15405 nm), operating over the range 10 < 2θ <85°, at a scanning rate of 5°/min and at a fixed incident angle (*ω* = 0.5°). JCPDS reference cards were used to identify the crystalline phase. 

The electrical characteristics of the MIS structures were measured by various methods at different temperatures and frequencies. The current–voltage (*I-V*) characteristics were measured at temperatures of 294 K and 77 K with a cycle sequence starting from 0 V toward negative or positive voltages up to maximal amplitude applied to the top Al–dot contact followed by a voltage reversal toward zero voltage. The duration of each measurement cycle was approximately 20–25 min. Room temperature 1 MHz capacitance–voltage (*C-V*) and conductance–voltage (*G-V*) measurements were conducted on a digital LCR meter E7-12. The impedance of these MIS structures was measured at room temperature with a Tesla BM-507 impedance meter applying test voltage frequencies in the range of 50–500 kHz. The conductance *G* and the capacitance *C* values were calculated by the expressions $G=\frac{\cos(\varphi_{m})}{\left|Z_{m}\right|}$ and $C=-\frac{\sin\left(\varphi_{m}\right)}{\omega \left|Z_{m}\right|}$, where │*Z_m_*│ is the measured impedance amplitude value and *φ_m_* is the phase angle at each test voltage frequency.

The gas-sensing measurements were performed under laboratory conditions (dry gases, in the absence of humidity) using an AC impedance spectrometer (Solartron 1260, AMETEK Inc., Berwyn, PA, USA) with an electrochemical interface. The AC bias amplitude applied was 1.5 V. The Electrochemical Impedance Spectra (EIS) were recorded in the frequency range of 100 Hz to 1 MHz from room temperature to 400 °C with a ProboStat cell (NorECs, Oslo, Norway) under a controlled atmosphere with a continuous gas flow of 177 mL/min. The sensor samples were in the form of standard microscope glass substrates, without additional metallic electrodes printed on the glass surface, cut to size to fit the sample holder of the sensing cell. The ProboStat cell was equipped with a 4-point probe kit to be compatible with this specific sample type (the platinum electrodes of the sensing cell were placed in direct contact with the sensitive film of the sensor sample). The electrical response of the sensitive films was measured for different CO concentrations (in the range of 0–2000 ppm), at a working temperature of 400 °C, without additional signal processing/filtering.

Photocatalytic tests were performed using 1 × 1.5 cm^2^ samples in 15 mL methylene blue solution (at the initial concentration of 10 ppm, according to the ISO 10678: 2010 standard [33]). The volume was chosen to allow for light penetration to the photocatalytic material, through no more than 2 cm of solution. To prevent pollutant solution evaporation during the tests, a quartz glass was placed on top of the quartz beakers containing the thin films and the pollutant solution. Additional distilled water (no more than a total of 2 mL over the 9 h testing period, per glass) was added dropwise, when necessary, to maintain a constant volume. Contact time was 1 h in the dark to achieve adsorption–desorption equilibrium, followed by 8 h irradiation using UV–Vis radiation (*G_total_* = 55 W/m^2^, *G_UV_* = 5 W/m^2^). A photo-reactor equipped with 2 UV light tubes (Philips, TL-D BLB 18 W/108, λ = 315–400 nm) and 5 Vis light tubes (Philips, TL-D Super 80 18 W/865, λ = 400–750 nm) (Koninklijke Philips N.V., Amsterdam, The Netherlands) was used.

Samples were taken every hour and the solution absorbance was determined using a Perkin Elmer Lambda 950 Spectrophotometer and the photocatalytic efficiency of the samples was calculated using the formula: $\eta =(A_{0}-A_{t})/A_{0}*100$, where *A*_0_—absorbance of the initial MB solution; and *A_t_*—absorbance of the MB solution after *t* hours contact with the photocatalyst.

To test the Vis-activation of the samples, tests under UV irradiation (*G_UV_* = 5 W/m^2^) were performed under similar conditions to those performed under UV–Vis irradiation. Additionally, the MB removal capacity of the photocatalyst through adsorption was evaluated by performing the same tests under no irradiation (in dark) for 9 h.

Photolysis experiments were developed under the same conditions, using UV–Vis irradiation and the MB solution.

Heterogeneous photocatalysis can usually be described by the Langmuir–Hinshelwood (L-H) model [34,35]. According to this, the reaction rate constant, *k* (min^−1^), can be determined as the negative slope of the linearization of ln(*C*/*C*_0_) as a function of time, where *C* is the concentration of the MB solution at different times during the experiment and *C*_0_ is the initial concentration of the MB solution.

## 3. Results and Discussion

### 3.1. XRD Data Analysis

**Figure 1:**
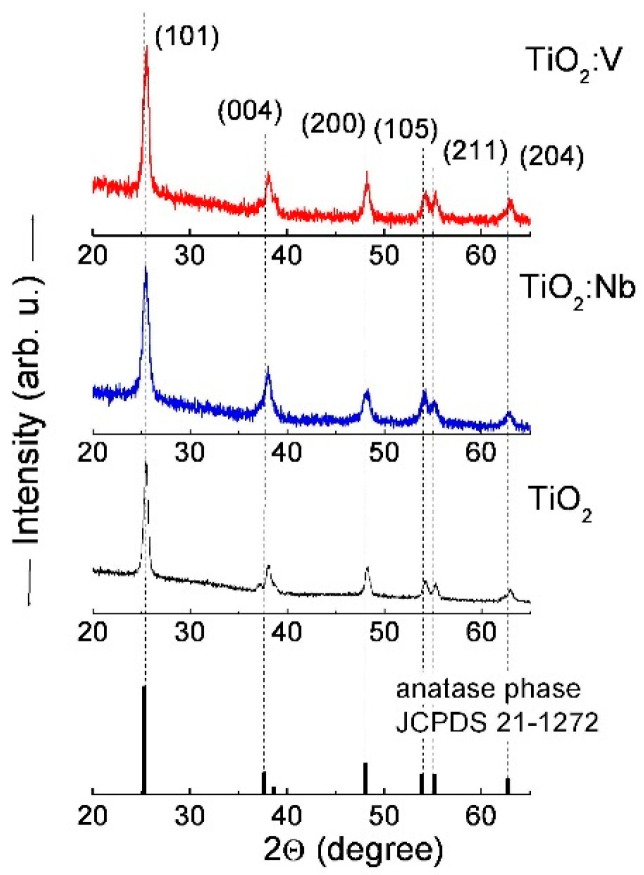
Fragments from XRD patterns of TiO_2_:Nb(V) multilayers illustrating the most intense Bragg peaks of the anatase TiO_2_ crystalline phase. The XRD spectrum of reference TiO_2_ from the card JCPDS of 21-1272 is also presented.

The XRD patterns of TiO_2_ and TiO_2_:Nb(V) samples are presented only in the spectral range of 20° < 2θ < 60° in Figure 1, where the more intensive peaks are situated. Here, we extended our earlier studies [28] by considering the lattice parameters in more detail and taking into account the influence of internal lattice strains, generated by the sol-gel technology, on the broadening of Bragg peaks.

The position of the peaks slightly varies related to the pure sol-gel TiO_2_ sample and they are situated as single peaks around ~25.5°, ~38.1°, ~48.2° and ~62.9° and as a double peak around ~54.2° and ~55.2°. All correspond to the TiO_2_ anatase phase with a tetragonal structure and I41/amd (141) space group according to the XRD reference card JCPDS 21-1272 [36]. The corresponding crystal planes of (101), (004), (200), (105), (211) and (204) are indicated in Figure 1. The broad peaks are evidence of the nanocrystalline structure with small-sized crystallites. Although no peaks related to Nb- or V-oxide phases were detected, the successful incorporation of these metal atoms was verified by XPS measurements, which indicated the presence of Nb^5+^ and V^5+^ states in the titania lattice [28,32]. 

The average size of nanocrystallites (*D*) was estimated by the Scherrer formula of D=(Kλ)/(βcosθ), where *β* is the full width of the peak broadening at half maximum (FWHM) after XRD baseline correction, *θ* is the angle of the Bragg peak position and *λ* is the X-ray wavelength of the Cu-Kα source. The particle shape factor or Scherrer’s constant is taken as K = 0.94 [37]. In addition, the size of nanocrystallites was also calculated from the other reflections with considerably broaden peaks with less than ~30% intensity. The results are summarized in Table 1. They point out that the introduction of V or Nb dopants in the TiO_2_ matrix reduced the crystallites’ size in comparison to that of sol-gel TiO_2_ sample. The average nano-crystallite size estimated from the most intense (101) Bragg peak was ~18.4 nm in the undoped TiO_2_, while it was ~14.5 nm in the V-doped TiO_2_ and ~13.9 nm in the Nb-doped TiO_2_. These are in good agreement with our TEM observations [29].

The lattice constants *a*, *b* and *c* are estimated using the Bragg law relation of sinθ=nλ/2d, where *d* is the interatomic distance (*d*-spacing) and *n* is integer for a given crystal plane. These constants were calculated for the (101), (004) and (200) reflections and the obtained values are given in Table 1. In the tetragonal crystals, *a* = *b* and the *d*-spacing is equal to 1/*d*^2^ = (*h*^2^ + *k*^2^)/*a*^2^ + *l*^2^/*c*^2^, where *h*, *k*, *l*, are the Miller indices of a crystal plane [37]. Therefore, *a* and *c* were calculated directly for the (004) and (200) reflections as 1/*d*^2^ = 16/*c*^2^ and 1/*d*^2^ = 4/*a*^2^ from the corresponding formulas *sinθ*_(004)_ = 2*λ*/*c* and *sinθ*_(200)_ = *λ*/*a*, respectively. The obtained values in the sol-gel samples are smaller compared to those of the reference TiO_2_ (JCPDS of 21-1272), which explains the observed shift in the Bragg peaks from the standard values.

**Figure 2:**
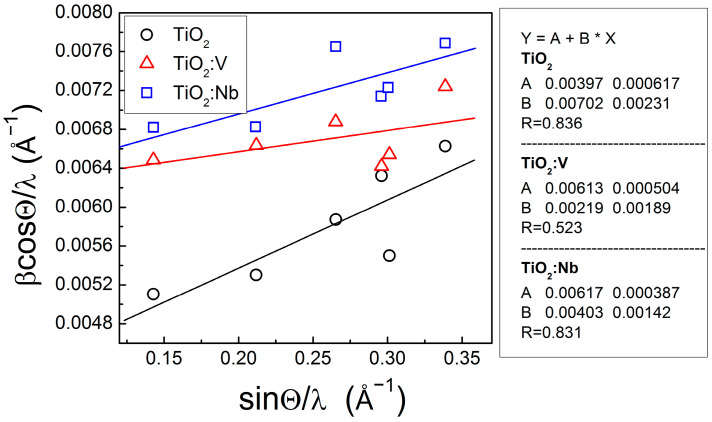
Williamson–Hall plots for the sol-gel pure and doped with Nb(V) TiO_2_ multilayers. The equation of the linear fit Y = A + B × X is inserted.

The observed alteration in the peak positions and lattice constants from the reference ones suggests that lattice strains may be expected in these samples. The existence of internal strains in the crystal lattice leads to diffraction peak broadening and introduces an error in β_FWHM_ when determining the crystallite sizes. Williamson and Hall proposed a method to separate the influence of lattice strain from the effect of grain size distribution in the Bragg peaks broadening [37,38]. The correlation between Bragg peak broadening caused by reducing particle sizes and average lattice strain can be expressed with a linear relationship *βcosθ*/*λ* = *K*/*D_e_* + *ηsinθ*/*λ*, where *D_e_* is the effective particle size and *η* is the effective strain [37]. We built the *β_hkl_ cosθ*/*λ* versus *sinθ*/*λ* plots (W-H plots) for the lattice planes of (101), (004), (200), (105), (211) and (204). By drawing the linear fit of the experimental points, the intersection of the line with the ordinate gives the effective crystallite size when lattice strain is zero and the slope of the line—the contribution of the effective internal strain to peak broadening. These plots are presented in Figure 2, where the uncertainties by the linear approximation are also given. 

The effective grain size *D_e_* in a sol-gel TiO_2_ free of lattice strains is 23.7 ± 3.2 nm, as derived from the extrapolation of the W-H plot. This value is bigger than that (18.4 nm) calculated from the (101) diffraction peak (Table 1), which proves the generation of structural strains during the growth of the layers by the given sol-gel technology. The corresponding effective grain sizes *D_e_* in the doped TiO_2_:V and TiO_2_:Nb samples are 15.33 ± 1.2 nm and 15.23 ± 1.0 nm, respectively.

The slope of the fitted lines is positive, indicating the presence of tensile lattice strains in all samples. Contrary to expectations, the doping of TiO_2_ layers with V or Nb atoms releases the strains to some extent, which leads to smaller values of the slope for TiO_2_:V and TiO_2_:Nb layers (Figure 2). From the lattice constants data in Table 1, one may suggest that the tensile strain is applied in the c-axis direction. The largest increase in the *c* lattice constant and the largest decrease in the *a* = *b* lattice constants are observed for TiO_2_:Nb films for which the effective strain is also higher. The tensile strain, estimated from the slope of the linear fit, is 7 × 10^−3^ ± 2.3 × 10^−3^ in the undoped TiO_2_ sample and 2.19 × 10^−3^ ± 1.9 × 10^−3^ and 4.07 × 10^−3^ ± 1.42 × 10^−3^ in the doped TiO_2_:V and TiO_2_:Nb, respectively. In statistical accounts the effective strain can be given as R^2^ × 100% and these values are 69.89%, 27.35% and 69% for TiO_2_, TiO_2_:V and TiO_2_:Nb, respectively.

The density of the unit cells was calculated from the relation of *ρ* = (*nM*)/*NV*, where *M* is the molecular weight of TiO_2_ (79.88 g/mol), and *N* is the number of Avogadro. The integer *n* is equal to the number of atoms per unit cell and in the case of anatase TiO_2_, it is four [39]. Further, the specific surface area *S_a_* was calculated by the formula *S_a_* = 6/(*D_e_ρ*). These values are also presented in Table 1.

The results in Table 1 show the weak variation in the structural parameters with doping. This supports the XRD observations that the TiO_2_ structures formed by the given sol-gel technology are very similar, indicating that V or Nb doping with low concentration (1.2 at. %) only slightly modifies the anatase TiO_2_ structure. Although the studied sol-gel samples have smaller lattice constants and correspondingly higher densities of unit cells compared to the reference TiO_2_ [36], they are well correlated with the results presented in the literature [40,41]. The smaller grain size and larger specific surface area of the nanocrystallites in the Nb(V) doped TiO_2_ samples are an advantage for sensor and photocatalytic applications.

The XRD studies show similar structures in these sol-gel layers, indicating the rather stable technological processes at their preparation. However, their optical and electrical properties may be different, as discussed below in the following sections.

### 3.2. SE Data Analysis

**Figure 3:**
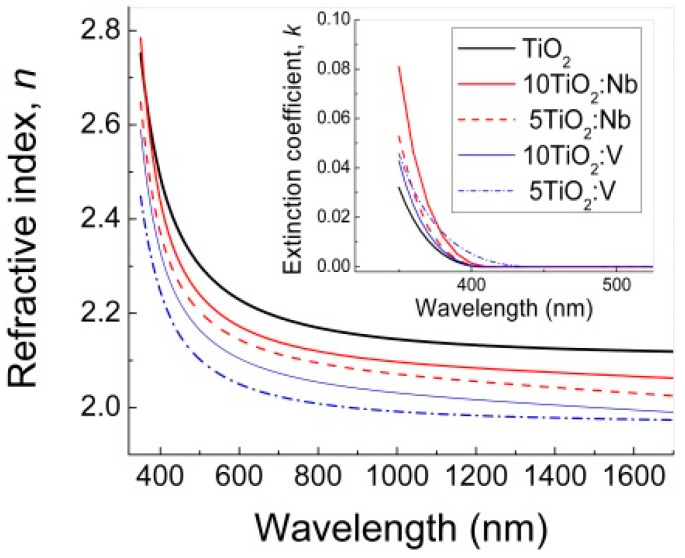
Dispersion curves of the refractive index *n* and extinction coefficient *k* (inserted) of pure and Nb(V) doped TiO_2_ multilayers.

**Figure 4:**
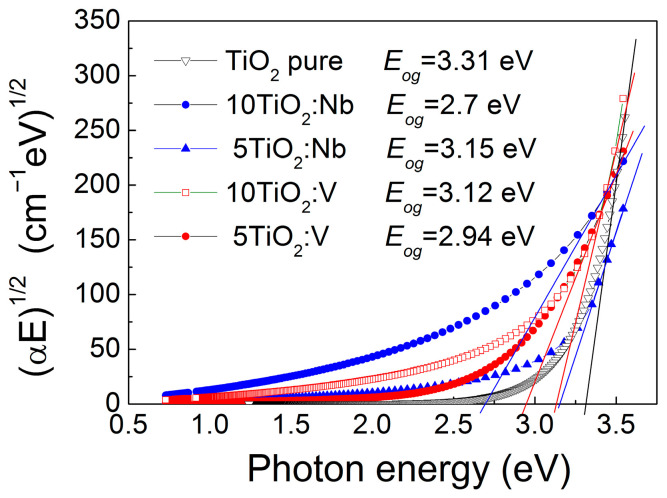
Tauc’s plots of pure and Nb(V) doped TiO_2_ multilayers. The optical bandgap energy values are inserted.

**Figure 5:**
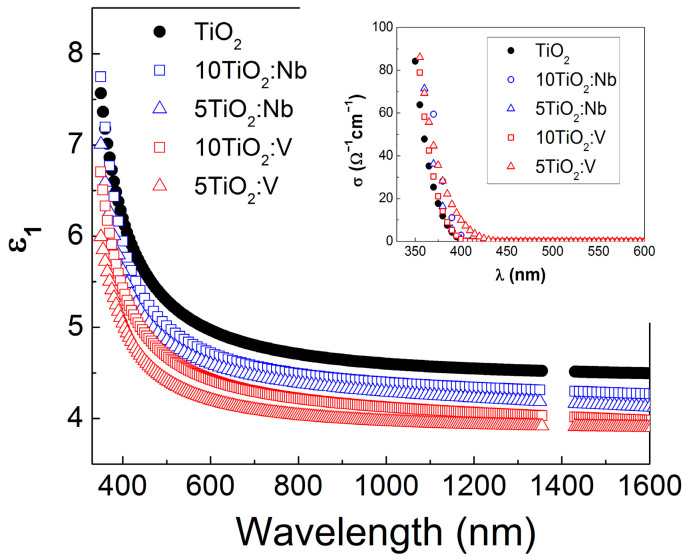
Optical dielectric constant ε_1_ versus wavelength *λ* of pure and Nb(V)-doped TiO_2_ multilayers. In the insert, the optical conductivity *σ* values versus *λ* for the studies films are presented.

The measured ellipsometric angles *Ψ*(*λ*) and Δ(*λ*) as a function of the wavelength *λ* were simulated with a three-layer optical model (top layer, representing the surface roughness, TiO_2_ layer and a native SiO_2_ (~1 nm) on c-Si substrate) and using the General Oscillator model containing one Tauc–Lorentz oscillator of corresponding software (supplied by J.A. Woollam Co. Inc.). In the SE fitting, the surface roughness of the films was considered an optical top-layer consisting of 50% voids and 50% TiO_2_, and was modeled by applying the Bruggeman’s effective medium approximation (BEMA) theory [42]. The quality of the fit, namely, the superposition between the experimental and calculated data, was assessed by the Mean Squared Error (MSE) procedure. From the analysis of the SE spectra, the optical constants, refractive index (*n*) and extinction coefficient (*k*) and the dielectric constants *ε*_1_ = *n*^2^ − *k*^2^ and *ε*_2_ = 2*nk* were evaluated. Knowing these quantities, the absorption coefficient (*α* = 4*πk*/*λ*) and optical conductivity (*σ* = *ε*_2_*ε*_0_*ω*, where *ε*_0_ is the vacuum absolute permittivity and *ω* is the wave frequency), were determined. The results are summarized in Figure 3, Figure 4 and Figure 5 and in Table 2, where the film thickness (*d_film_*) and an optical top layer, representing the surface roughness (*d_rough_*), as well as the porosity (*P*) of the films are also included.

Our earlier SEM and AFM studies [28,32] showed uniformly distributed shallow cavities on the surface of the sol-gel layer. Because the films are grown using a layer-by-layer technique, such cavities become visible by SE as pores in the films’ volume and are given as porosity values in Table 2. From Table 2, it is clearly seen that in the pure TiO_2_, the porosity is very low, while in the doped layers, it increases sharply in the 5-layered TiO_2_:Nb(V) films, and to a lesser extent, in the 10-layered TiO_2_:(Nb(V) films. The corresponding values of these quantities for the films deposited on glass substrates (not shown here) follow the same tendency observed for the films on silicon substrates. In the case of glass substrates, slightly larger thickness and porosity values are observed, suggesting the faster growth of sol-gel layers when amorphous microscopic glasses are used instead of c-Si ones. 

In Figure 3, the pure TiO_2_ film exhibits the highest *n* value. An increase in *n* value is also observed with the increase in the number of layers for the doped TiO_2:_Nb(V) films. This effect can be attributed to the denser structure in undoped TiO_2_ and the increase in porosity by introducing Nb(V) atoms into the doped TiO_2_:Nb(V) layers (Table 2).

The extinction coefficient values became negligible above λ~450 nm, testifying for good transparency in the visible spectral range (Figure 3). For the doped layers, the dispersion curves moved toward smaller energies (longer wavelengths), suggesting a narrowing in the TiO_2_ energy bandgap, *E_og_*. Considering that indirect electron transitions occur, the *E_og_* was evaluated from the absorption coefficient *α* (*α* = 4*πk*/*λ*), building the Tauc’s plots (*αE*)^1/2^ versus photon energy *E* and extrapolating the linear part of the curves toward zero absorption [43]. The interception of this line with the *E*-axis provided the *E_og_* values with an accuracy of ±0.05 eV. The corresponding Tauc’s plots are given in Figure 4. 

In all cases, the effect of doping is reflected in a decrease in the bandgap energy values compared to the pure TiO_2_ films (Table 2). The energy bandgap of pure 5-layered TiO_2_ is 3.31 eV, while for the 10-layered TiO_2_, it is 3.28 eV, as the observed difference in the *E_og_* values is in the frame of accuracy of ±0.05 eV. The introduction of Nb with 5+ valence state in the titania lattice creates defect levels in the TiO_2_ energy bandgap, which leads to the reduction in the *E_og_* value to 3.15 eV in the 5TiO_2_:Nb film. In the 10TiO_2_:Nb film, this effect is stronger because the thicker the layer, the more defect centers are created, leading to a further reduction in *E_og_* down to 2.7 eV. Moreover, the higher internal strains observed in the Nb-doped TiO_2_ layers may also contribute to the *E_og_* narrowing in these films. Such an effect of strain on *E_og_* has been reported in the literature [44]. The smaller change in *E_og_* of 5- and 10-layered TiO_2_:V films is due to the fact that V doping is accompanied by the formation of V_2_O_5_ oxide phase at the grain boundaries, which reduces the efficiency of V doping [45].

In general, higher porosity offers a larger active surface area when films are applied for gas detection or pollutant photodegradation from water. In this sense, the increased porosity of the Nb(V)-doped TiO_2_ in comparison to the pure sol-gel TiO_2_ (Table 2) is expected to lead to an improvement in sensor and photocatalytic properties. Since the V-doped TiO_2_ films show the highest porosity values, we might expect an enhanced response in CO sensing.

### 3.3. Electrical Characteristics of MIS Structures

For an effective use of sol-gel TiO_2_:Nb(V) in a proper application, it is necessary to understand the conduction mechanism and the way in which deposition conditions influence the generation and distribution of the defects states in the studied films. Smaller crystallites in the Nb(V) doped sol-gel TiO_2_ films, as the XRD data analysis revealed, suggest more grain boundaries and thus a stronger influence on charge carriers transport properties. 

In this Section, we present a comparative study of the electrical properties of multilayered TiO_2_:Nb(V), linking the role of deep levels to point defects to their properties, particularly to the electron charge transport mechanism. In our earlier studies on TiO_2_:Nb films, the electrical characteristics were recorded only at room temperature [29], while in [30], the studies were conducted only on 5-layered TiO_2_:V multilayers. Here, we extended the studies on 5TiO_2_:Nb(V) and 10TiO_2_:Nb(V) films, built in MIS structures, and the electrical properties of these MIS structures were studied by I-V, C-V, G-V and impedance measurements in different temperatures and frequencies in order to determine the main electrical parameters and from them, to draw some conclusions regarding the charge transport mechanism.

**Figure 6:**
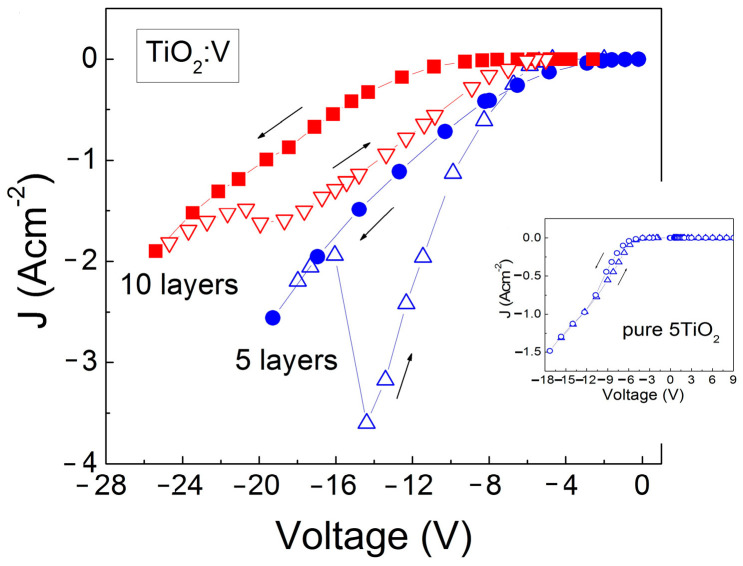
J-V characteristic of the MIS structure with 5TiO_2_:V and 10TiO_2_:V. In the insert, the J-V curves of pure 5TiO_2_ is presented.

In Figure 6, the *I-V* characteristics of V-doped TiO_2_ films are illustrated as the current density *J* = *I*/*s* is plotted against the voltage applied to the Al-dot contact on the oxide surface. The current at a positive voltage remains consistently below 10^−7^ A, giving rise to a strong asymmetry between the *J* values at positive and negative voltage values. A similar behavior was registered in the pure TiO_2_ (inserted in Figure 6) and Nb-doped TiO_2_ films as well.

At the Si-TiO_2_:V interface, a p-n heterojunction has been formed by electron diffusion from the V-doped n-type TiO_2_ layer to the p-type Si substrate. At the Si-TiO_2_ interface, the positive charges of V donors in TiO_2_ and the negative charges of the electrons diffused into Si substrate generated an electric field. This built-in electrical field creates an energy barrier, *qφ_b_*, for electrons moving from the TiO_2_ film toward the Si substrate and forming a space charge layer at the Si–TiO_2_ interface. Negative voltage (−*V*) at the Al-dot contact creates an electrical field which opposes the built-in electrical field at the interface and, therefore, the electron energy barrier at the Si-TiO_2_ interface decreases to *q*(*φ_b_* − *V*). However, at negative voltages, the holes in p-Si do not participate in the current because for them, the energy barrier at the interface is high (~2 eV). When positive voltage (+*V*) is applied to the Al-dot contact, the created electrical field is in the same direction as the built-in electrical field at the Si-TiO_2_ interface and therefore, the electron energy barrier at the Si-TiO_2_ interface increases to *q*(*φ_b_* + *V*). These changes in the electron energy barrier at the interface by positive and negative voltages explain the observed asymmetry of the current in the TiO_2_:Nb(V) structure, which is in the 10^4^–10^5^ range.

The presence of counterclockwise hysteresis in the V-doped TiO_2_ films (Figure 6) in the forward direction suggests that charges are captured at deep levels in the oxide with time constants higher than the time needed for measuring the initial and return stages. The captured charges increase the current in the return stage of the *I-V* measurement. A similar but smaller displacement of the subsequent branch from the initial one in the hysteresis curve was observed in the TiO_2_:Nb films.

**Figure 7:**
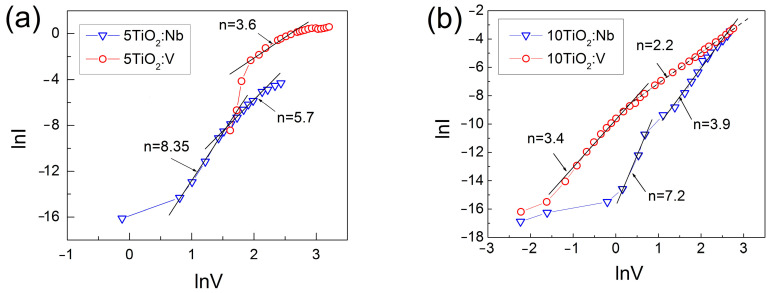
Logarithmic representation of I-V dependence of MIS structures with the 5-layered (**a**) and 10-layered (**b**) TiO_2_:Nb and TiO_2_:V films.

The specific resistivity, *ρ_f_*, was calculated from the *I-V* characteristics, measured under the forward bias at higher voltages, from the resistivity *R* being equal to *R* = Δ*V*/Δ*I* and *ρ_f_*_=_ *Rs*/*d_f_*, where ∆*V* and ∆*I* are the differential voltage and current values, *d_f_* is the film thickness, and *s* is the dot contact area. The calculated *ρ_f_* values are given in Table 3. As can be seen, *ρ_f_* is in the order of 10^4^ Ωcm. Such large specific resistivity values can be a result of charge compensation taking place, as previously reported by Baumard et al. [46]. The high resistivity values, as well as the implied presence of charge traps in the film, indicate that space charge limited current occurs. This can be checked by determining the value of *n* from the *I~V^n^* dependence. By plotting the *lnI* versus *lnV* graphs (Figure 7), the obtained *n* values are higher than 2, regardless of the dopant type used. Such values of *n* indicate that the forward current is space charge-limited current via deep levels with different energies in the TiO_2_:Nb(V) energy gap.

**Figure 8:**
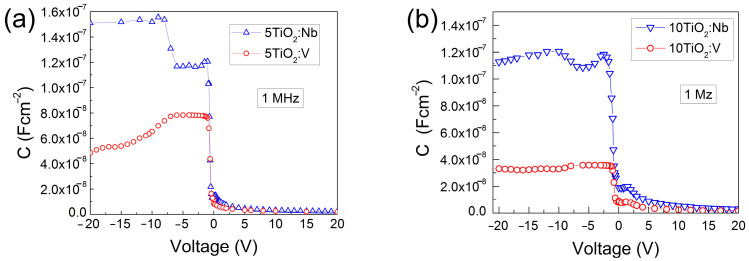
*C-V* characteristics of MIS structures with 5-layered (**a**) and 10-layered (**b**) TiO_2_:Nb(V) films.

The 1 MHz *C*-*V* characteristics are presented in Figure 8. In all cases, at a reverse direction, when positive voltage is applied to the oxide surface, the capacitance values decrease, indicating that the thickness of the space charge layer formed at the Si-TiO_2_:Nb(V) interface increases at these voltages. For negative voltages (forward direction), the thickness of the space charge layer decreases, which is reflected in the increase in the measured capacitance at these voltages.

The injected positive charge Δ*Q_i_*_+1,*i*_ at the edge of the space charge layer under the change in applied forward bias voltage Δ*V_i_*_+1,*i*_ = *V_i_*_+1_*−V_i_* is given by the following formula:

Δ*Q_i_*_+1,*i*_ = (−C*_m_*_,*i*+1_*V_i_*_+1_ + *C_m,i_V_i_*)/*s*, (1)

where *C_m_*_,*i*+1_ is the measured capacitance at the *i* + 1 step and *s* is the contact area. The corresponding decrease in the space charge layer thickness *Δw_i_*_+1,*i*_, is given by the following formula:

Δ*w_i_*_+1,*i*_ = sε_Si_ [(1/*C_m_*_,*i*+1_) – (1/*C_m,i_*)], (2)

By dividing Δ*Q_i_*_+1,*i*_ by Δ*w_i_*_+1,*i*_, one may estimate the density of diffused electrons $n_{e}$ between deep levels with distances *w_i_* and *w_i_*_+1_. The highest reliable values of *n_e_* for the TiO_2_ structures are calculated around the forward bias voltage of −1 V. The estimated *n_e_* values are in the order of 10^16^ cm^−3^. The charge of these diffused electrons partly compensates the positive charge of dopants and, thus, the real value of the donor doping level *N_d_* in the bulk of the TiO_2_Nb(V) films is higher than *n_e_* but close to it. In spite of the high doping values of the TiO_2_ films (*N_d_* > *n_e_*), the specific resistivity remained considerably high, indicating the presence of charge compensation.

By using the resistivity and carrier concentration values, the electron effective mobility *µ_eff_* values are estimated by the expression *ρ_f_* = 1/(*qn_e_μ_eff_*). The obtained *µ_eff_* values are in the range of 10^−3^–10^−4^ cm^2^V^−1^s^−1^, being lower compared to those previously reported by other groups [47,48]. 

The above determined electrical parameters of the multilayered TiO_2_:Nb(V) films are also summarized in Table 3.

**Figure 9:**
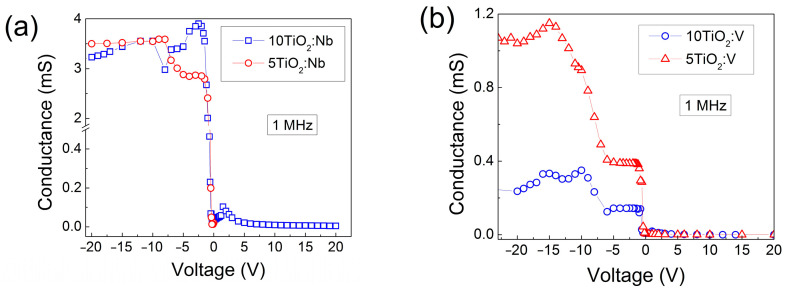
G-V characteristics of MIS structures with TiO_2_:Nb (**a**) and TiO_2_:V (**b**) films.

The variation in the capacitance trend (bump appearances) at negative voltages (below −3 V) in the TiO_2_:Nb films (Figure 8) is an indicator of the existence of deep levels in the film volume and/or at the Si-TiO_2_ interface. Since the *G-V* measurements are more sensitive to the appearance of deep levels, their presence can be clearly observed in the *G-V* characteristics of the TiO_2_:V MIS structure (Figure 9) even if no bump could be detected at negative voltages in the *C-V* characteristics of the same MIS structures (see Figure 8).

By using the maximum peak value *G_max_*, taken from the *G-V* curve, and the angular frequency *ω*, the additional capacitance of the heterostructure caused by the captured charges in deep levels can be calculated using the expression Δ*C*(*V*) = *G_max_*/*ω*. If the levels are situated at the interface between the substrate and oxide film, then the sheet density of deep levels *N_SS_* is equal to *N_SS_* = Δ*C*/*qs*. When the deep levels are situated in the oxide bulk, the energy density of traps *N_tb_* is calculated as *N_tb_* = *N_SS_*/*d_f_.*

Depending on the voltage value at which these conductance variations take place, and on the film thickness, the energy density of traps is different. This suggests that the defect-related energetic levels are present at different positions in the TiO_2_ bandgap. As a general observation, the concentration of defects is higher for the Nb-doped films compared to the V-doped TiO_2_ (Table 3), showing that the incorporation of Nb in the TiO_2_ matrix yields a more defective structure. This is in good correlation with the observed higher porosity values in TiO_2_:Nb films given in Table 2. Moreover, the 5-layered doped TiO_2_ samples exhibit higher *N_tb_* values compared to their 10-layered counterparts, for which the porosity values are also larger. For all samples studied, however, the presence of acceptor defects in the films, either at the interface or in the bulk, leads to compensation of the introduced donors in the TiO_2_ films, resulting in the observed high resistivity despite the doping.

To reveal the charge transport mechanism, admittance measurements in the 50 Hz–500 kHz range were performed on the investigated Si-TiO_2_:Nb(V) MIS structures without applying bias voltage. The real and imaginary parts of the measured impedance vary considerably with the variation in the test voltage frequency. These variations are connected with the changes in the conductance and capacitance of the Si-TiO_2_:Nb(V) MIS structures. The sharp increase in capacitance at lower frequencies below 100 kHz (not shown here) indicates that the deep levels have different response times, with the time constants being longer than 80 µs. This behavior is present in all doped TiO_2_ films, regardless of dopant type or film thickness.

**Figure 10:**
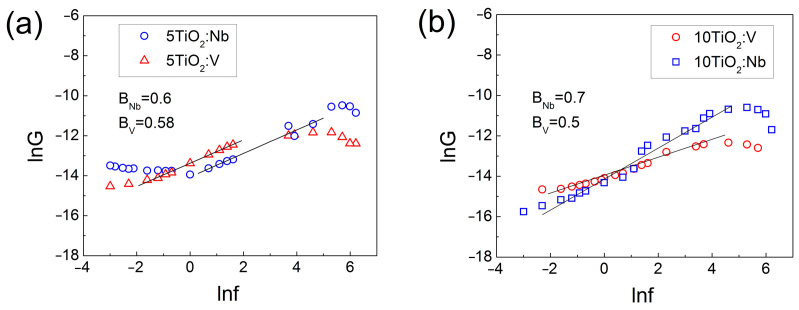
ln*G* versus ln*f* plot of MIS structures with 5TiO_2_:Nb(V) (**a**) and 10TiO_2_:Nb(V) (**b**) films. The frequency values are in kHz.

By plotting the *G-f* dependence as ln*G* against ln*f* (Figure 10), it is evident that the *G* values depend on the frequency in accordance with the Jonscher universal power law relation [49] for AC conductance, *σ_f_* = *σ_o_* +*A*(*2πf*)*^α^*, where α is the power law exponent. In the medium range frequency, where the graph is linear, the slope of the dependences varies between 0.5 and 0.7 for both Nb-doped and V-doped TiO_2_ thin films. Such a conductance–frequency dependence is consistent with the hopping of charged carriers from one occupied deep level to another unoccupied one [50]. A similar *G-f* dependence has been also observed for electron tunneling from occupied deep level to the nearest unoccupied one [51]. In order to clarify what is the nature of the electron transport mechanism in the studied TiO_2_:Nb(V) films, the *I-V* characteristics of the MIS structures with the Nb(V) doped TiO_2_ films were measured at 77 K and 294 K. From these, the energetic position of deep levels in the TiO_2_ energy bandgap and the distance between these levels were estimated.

**Figure 11:**
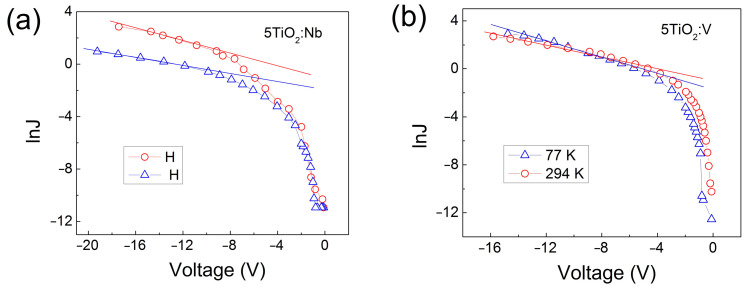
Forward *I*-*V* characteristics of MIS structures with 5TiO_2_:Nb (**a**) and 5TiO_2_:V (**b**) films given as ln*J* versus applied voltage V plots, measured at different temperatures.

In Figure 11, the temperature dependence of the *I-V* characteristics, exemplified for the five-layered TiO_2_:Nb(V) films, is given as ln*J* versus *V* plots in the forward direction (with negative voltages at the Al dot contact). 

For V-doped films (Figure 11b) the current densities at both temperatures are similar, indicating that the electron transport in these films is tunneling-type conduction. The same mechanism is valid for the Nb-doped films but only down to −5 V (Figure 11a). Below −5 V, the conductivity at 294 K becomes greater than that at 77 K. This points out that thermally activated conduction occurs in the Nb-doped layers under these conditions. In the case of thermally activated conduction, electrons hop from occupied energy levels to adjacent empty ones.

It can be seen that in the interval −9 and −16 V, the current density is almost linear and can be expressed as *y* = *a* + *bx*, i.e., ln*J* = *a* + *bV*. Using the expression ln(*J*_294_/*J*_77_) = *qφ_a_*[(1/*k*77) − (1/*k*294)], the activation energy of the current *qφ_a_* in these TiO_2_:Nb(V) MIS structures can be calculated. 

For the 5TiO_2_:Nb (Figure 11a), the activation energy is equal to *qφ_a_* = 17–19 meV, while for the 5TiO_2_:V (Figure 11b), it is equal to *qφ_a_* = 14–19 meV, which is definitively lower than the room temperature value of *kT*~25 meV and, therefore, the main contribution to the electron transport at both 77 and 294 K temperatures is accomplished by the electron tunneling from the occupied levels to the nearest empty ones in the TiO_2_ energy bandgap; in other words, the electrons move by inter-trap tunneling. 

In the case of inter-trap tunneling, the current density *J* is given by the following formula:

*J* = *J*_0_.*sinh*[*B*(*V* − *V_fb_*)], (3)

where *J*_0_ = *2qν*$\frac{\exp[\frac{-2{\left(2m*q\right)}^{1/2}\varphi_{t}^{1/2}w}{\hbar }]}{w^{2}}$; *B* = $\frac{{\left(2m*q\right)}^{1/2}w^{2}}{\hbar \varphi_{t}^{1/2}d_{f}}$; *ν* is the electron attempt to escape frequency from deep levels; and *d_f_* is the film thickness [52]. The electron effective mass, m*, is taken as equal to *m_e_* [53]. The value *ν* = 2.4 × 10^13^ s^−1^ of the electron attempt to escape frequency is estimated from the relation *hγ* = *kT_D_*, [54], where the Debye temperature, *T_D_*, for anatase TiO_2_ is 520 K and all other symbols have their common meaning. From the *C-V* characteristics in Figure 8, it is evident that the flatband voltage, *V_fb_*, is close to zero. This is evidence of a high-quality Si-TiO_2_ interface demonstrated by our TEM [29] and SEM [28] imaging. From Equation (3), it follows that extrapolating the linear part of the plot ln*J* versus *V* toward *V* = 0, from the slope *b* of the line and the intersection *a* with the *lnJ* axis, the mean distance *w* between deep levels situated around the Fermi level and the energy position of these levels *qφ_t_* in the TiO_2_ energy bandgap, respectively, can be calculated.

For the 5TiO_2_:Nb films, from the *I-V* measurements at 77 K the calculated mean values for *w* and *qφ_t_* are *w* = 2.09 × 10^−7^ cm and *qφ_t_* = 0.81 eV, respectively, while from the *I-V* measurements at 294 K, they are equal to *w* = 2.45 × 10^−7^ cm and *qφ_t_* = 0.54 eV. Having the *w* value, the concentration *N_t_* of these localized electron states is estimated by the relation *N_t_* ≈ 1/w^3^. In the 5TiO_2_:Nb films, it is equal to *N_t_* = 1.1 × 10^20^ cm^−3^ at 77 K and to *N_t_* = 6.8 × 10^19^ cm^−3^ at 294 K. The energy position *qφ_t_* = 0.81 eV of these levels at 77 K is close to the value of 0.8 eV for deep level in rutile TiO_2_, reported in [55]. The smaller value of *qφ_t_* = 0.54 eV at 294 K is connected with the contribution of thermally activated hopping, added to the total current. 

In the case of 5TiO_2_:V films, the parameters of the deep levels are calculated as described above. The values of *w* and *qφ_t_* are as follows: *w* = 3.27 × 10^−7^ cm and *qφ_t_* = 0.31 eV for measurements at 77 K; and *w* = 2.83 × 10^−7^ cm and *qφ_t_* = 0.38 eV for measurements at 294 K. From the relation *N_t_ ≈* 1/*w*^3^, the concentration of these localized electron states, *N_t_*, is estimated to be equal to *N_t_* = 2.86 × 10^19^ cm^−3^ at 77 K and equal to *N_t_* = 4.41 × 10^19^ cm^−3^ at 294 K.

When the values of *w* and *qφ_t_* are known, it is possible to estimate the rate of electron tunneling, *r_tun_*, from one occupied acceptor trap to the nearest unoccupied one by the expression *r_tun_* = $\nu \exp[\frac{-2{\left(2m*q\phi_{t}\right)}^{1/2}w}{\hbar }]$ and to compare it with the rate of electron hopping over the energy barrier *qφ_t_* between occupied and the nearest unoccupied one, *r_therm_* = $\nu \exp[-\frac{q\phi_{t}}{kT}]$. The ratio of *r_therm_*/*r_tun_* for the Nb-doped TiO_2_ film at 294 K is equal to 0.0573, while it is 2.26 × 10^−45^ at 77 K. From this, it follows that thermally activated hopping of electrons takes place at 294 K, while at 77 K, the current is exclusively a tunnelling one. At lower temperatures, any thermally activated process (hopping or emission to the TiO_2_:Nb conduction band) is excluded. This explains the higher values of the measured current at 294 K and at a given voltage in comparison with the measured current at the same voltage at 77 K. These results are in accordance with the variable range hopping at temperatures above 325 K and temperature-independent conductivity at temperatures below 200 K observed in Nb-doped TiO_2_ prepared by RF sputtering [56].

The calculated concentration of deep levels, responsible for the inter-trap tunneling in the TiO_2_:Nb and TiO_2_:V films, calculated at both temperatures, is in the 10^19^ cm^−3^ range. These deep levels are acceptor-type and they compensate the Nb or V donors. The degree of this compensation can be estimated from the ratio of net un-compensated (*N_d_ − N_t_*) concentration to the acceptor deep level concentration. As is reported in [57], when Nb-doped TiO_2_ films are prepared in oxygen containing ambient, the Nb donors are compensated, while if they are prepared in a reducing atmosphere, then the Nb donors are not compensated. This behavior of Nb-doped TiO_2_ films prepared in oxygen-rich environments has been explained by introducing oxygen interstitials in the TiO_2_ lattice, which act as acceptors and which can be eliminated by subsequent annealing in hydrogen [58]. Since the present sol-gel Nb or V-doped TiO_2_ films are prepared and annealed in oxygen rich ambient, one may expect the high degree of Nb or V compensation in the studied TiO_2_:Nb(V) films.

From the measured *I-V*, *C-V* and *G-V* characteristics, the concentration of donor-compensating defects in these films were estimated. Although the Nb- and V-doped TiO_2_ films possess excellent transparency (~80% in the Vis range [28]) and good anatase crystallinity, as well as successful doping (≥10^16^ cm^−3^) (Table 3), because of the low electron mobility (Table 3), they cannot be used as transparent conductive oxide (TCO) materials, unless the concentration of compensating acceptor levels, generated in the TiO_2_ energy gap during the TiO_2_ film preparation, is greatly decreased. On the other hand, nanostructured TiO_2_ films with such small grains (13–19 nm) possess indisputable advantages for their application as gas-sensitive films in the detection of various gases.

### 3.4. Gas Sensing Properties

**Figure 12:**
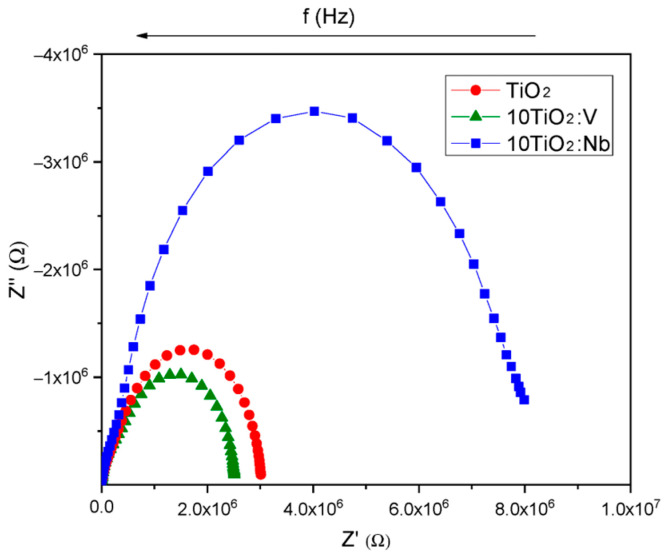
Nyquist plots for TiO_2_, 10TiO_2_:V and 10TiO_2_:Nb sensor samples at T_w_ = 400 °C, in dry air (carrier gas only).

**Figure 13:**
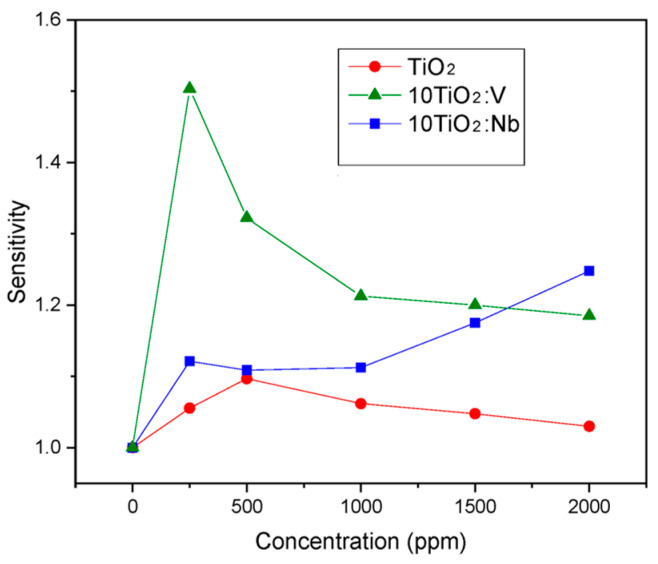
Sensor samples sensitivity at T_w_ = 400 °C, for different tested CO concentrations.

**Figure 14:**
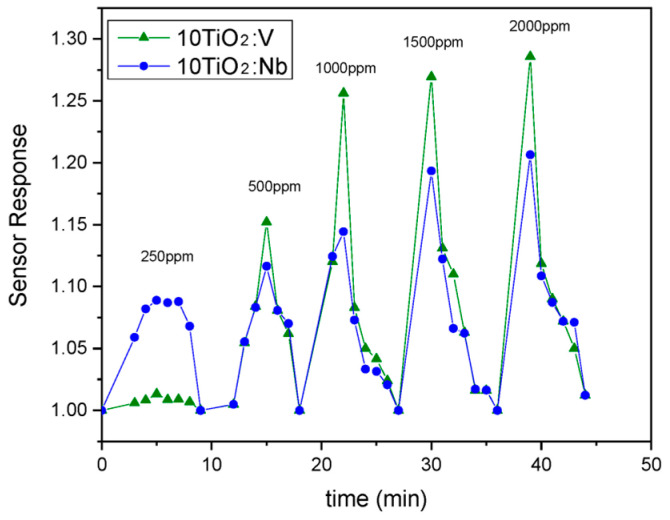
Response/recovery characteristics for 10TiO_2_:V− and 10TiO_2_:Nb-based sensors, T_w_ = 400 °C, for different tested CO concentrations.

As the results from Section 3.1 and Section 3.2 showed, the sol-gel TiO_2_:Nb(V) films are porous and crystallized in anatase phase, and, hence, they can be used for gas-sensing applications. The Nb and V dopants play multiple roles in this case: they hinder the phase transformation from anatase to rutile, act as a donor, narrow the bandgap and lower the electrical resistance, as showed by the above presented results. We conducted complex impedance measurements aiming to understand the mechanism of gas/solid interactions and to determine the active regions in the films (surface, grain and grain boundaries) that are involved in the detection of the analyzed gas. The measured impedances Z = Z′ + j Z″ (Z′ and Z″ being the real and imaginary components, respectively) were represented using Nyquist plots (Z″ versus Z′). Figure 12, Figure 13 and Figure 14 were plotted using the experimental recorded signals in their raw form, without additional filtering and/or simulation. All samples were tested under laboratory conditions (dry carrier and target gas; the humidity in the sensing chamber/sensor sample was eliminated by heating to the corresponding T_w_ under carrier gas flow for 1 h).

In Figure 12, the response of the sensitive films to CO is represented as Nyquist plots for TiO_2_, 10TiO_2_:Nb and 10TiO_2_:V at 400 °C in dry air (carrier gas only). The Nb-doped TiO_2_ films have the highest electrical resistance in air, followed in order by pure TiO_2_ and V-doped TiO_2_ films. 

After testing the doped/undoped sensitive films deposited on the microscope glass substrate (without additional metallic printed circuitry—referred to as “sensor samples”) in carrier gas only, at the optimum working temperature (T_w_ = 400 °C), the next step was to test their response under target gas, in our case CO. The tested target gas concentrations were in the range of 0–2000 ppm, thus covering the CO exposure limits from mild symptoms due to gas poisoning to lethal dosage (Table 4).

The sensor response (*S_r_*) was defined as the ratio between the electrical resistance of the film in the carrier gas (*R_air_*) and the electrical resistance of the film in the atmosphere containing the target gas (*R_CO_*), as seen in Equation (4).

(4) $$S_{r}=\frac{R_{air}}{R_{CO}}$$

According to the experimental results presented in Figure 13, the sensors based on V-doped TiO_2_ films exhibited the highest sensitivity to CO at the specified working temperature. Both doped films were more sensitive to CO than pure TiO_2_; so, the introduced dopants clearly promote gas sensing. Sensor response (*S_R_*) in all cases was small in value (*Sr_ma_*_x_ = 1.5), but this is due to the lack of imprinted metallic electrodes on the surface of the glass substrate. Additional metallic (Au or Pt) circuitry, usually in an interdigital form (IDE), is a key feature that greatly improves the charge transfer in the sensor structure leading to higher sensor response values [63]. Further studies using these films compositions will be performed in the future using an IDE-based chemiresistor support.

The response recovery characteristics of the Nb-doped TiO_2_-based sensor show a sharp response (*t_resp_* = 5 min) and a complete sensor recovery (*t_rec_* = 5 min) after sensor exposure to different CO concentrations (in air) in the range of 0–2000 ppm at a sensor operating temperature of T_w_ = 400 °C. The sensor response value increases linearly with increasing target gas concentration; so, the maximum tested concentration is not an actual detection limit for the sensor, but testing beyond the 2000 ppm value is pointless, according to the exposure limits specified in Table 4. On the other hand, comparable response recovery characteristics were obtained for the 10TiO_2_:V sensor sample, under similar testing conditions, indicating that *S_r_* values are a bit higher comparing to those obtained for the Nb-based sensor, which is in good correlation with the sensitivity data showed in Figure 13. The tested sensor responded sharply, as in the previous case, when exposed to CO-containing atmospheres (*t_resp_* = 5 min) and sensor recovery was complete (*t_rec_* = 5 min). Figure 14 shows which CO sensor performed best under similar testing conditions. Although the Nb-based sensor performed better at lower concentration (250 ppm CO in air), the V-based sensor performed better overall. 

The sensing mechanism is a two-stage process. In the first stage, as the samples are placed in the dry carrier gas only (air), oxygen species O_2_^−^, O^−^ or O^2−^ will form, extracting electrons from the valence band of the film and creating a depletion layer. In the second stage, the target gas (CO, a reducing gas) will react with the charged oxygen species, releasing electrons back to the sensitive film during the formation of CO_2_ molecules. The sensing process is optimum at a working temperature of 400 °C, as proven in our previous work [32].

The role of Nb and V dopants is that of donors in the TiO_2_ matrix as they insert electrons into the conduction band and increase the number of available charge carriers, which will promote more reaction sites for CO oxidation than in the case of the undoped TiO_2_ [32]. Thus, both doped sensors have improved sensing performance compared to the sensor based on pure TiO_2_ films. It should be emphasized that V doping of the TiO_2_ films leads to better overall CO-sensing results. This fact also correlates with the improvement in the resistance baseline values obtained at the operating temperature of the sensor in carrier gas only (Figure 12).

The low *S_r_* values, yielded by all tested doped sensors, are compensated by the low concentration of dopants used. The thin films are deposited via an environmentally friendly technique, combined with the simple sensor design (standard microscope glass substrate) without a standard Pt/Au printed circuit and the relatively low sensor operating temperature (400 °C), in comparison with those obtained by other research groups on similar sensitive films compositions [64,65]. These advantages may be employed for large-scale fabrication of a low-budget carbon monoxide detector.

The state of the art shows that although Niobium has previously been used for doping TiO_2_ films, with good results in CO detection [64,65,66,67,68,69], the literature on Vanadium doping of TiO_2_ for such an application is scarce [70]. The data findings presented in this section, regarding this particular aspect, can be considered a new achievement.

### 3.5. Photocatalytic Properties

**Figure 15:**
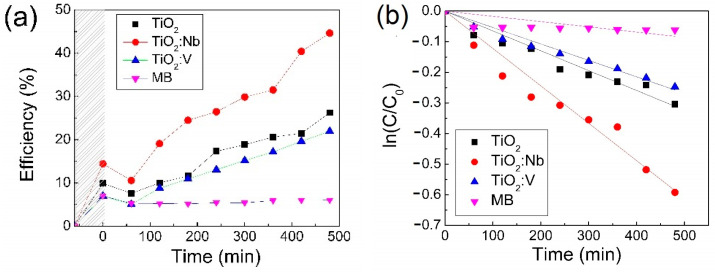
Photocatalytic efficiency in methylene blue solution (**a**) and linearization of ln(*C*/*C*_0_) against time (**b**) for the 5TiO_2_, 5TiO_2_:Nb and 5TiO_2_:V thin films deposited on microscopic glass.

The photocatalytic efficiency of the 5TiO_2_, 5TiO_2_:Nb and 5TiO_2_:V samples was determined under UV–Vis radiation with low-irradiance and the results are presented in Figure 15. As can be seen, all samples are photoactive under UV–Vis irradiation. The relatively low photocatalytic efficiency of the samples can be correlated with the low irradiance value (55 W/m^2^). This leads only to a small number of electron–hole pairs being formed and thus the amount of HO• radicals obtained is limited. However, it is expected that under irradiation with a higher irradiance value (e.g., sunlight), the photocatalytic efficiency will also increase, as the oxidative species’ number increases. Moreover, some compensation of the dopant injected electrons into the TiO_2_ matrix by the acceptor levels, as identified from the electrical measurements, can also limit the photocatalytic efficiency of the doped samples.

By looking at the trend of the variation in the photocatalytic efficiency with time, it can be seen that none of the studied samples reach a plateau and, therefore, the clogging of the sample surface with pollutant or by-product molecules is limited. This is very promising for upscaling. Moreover, it can be seen that, when using the Nb-doped TiO_2_, the photocatalytic efficiency nearly doubles compared to that of pure TiO_2_, which can be due to the extension of the activation range of TiO_2_ from the UV to the Vis domain due to doping (as confirmed by the SE measurements with TiO_2_ *E_og_* = 3.31 eV and TiO_2_:Nb *E_og_* = 3.15 eV). It is expected that the TiO_2_:V sample, which has the lowest bandgap energy (*E_og_* = 2.94 eV for TiO_2_:V), will exhibit the highest photocatalytic efficiency. However, as seen from Figure 15, the Vanadium-doped thin film is less efficient than the Niobium-doped film. This is in good agreement with the gas-sensing performance of the samples as well, where Nb-doping led to a higher formation of oxygen species, compared to undoped TiO_2_ and TiO_2_:V. Although the gas-sensing tests were performed on 10-layer thin films, the trend is expected to be similar for the 5-layer samples. It is known that superoxide radicals can contribute to the photodegradation of the organic pollutants and, therefore, this may explain the better photocatalytic activity of TiO_2_:Nb. 

Another reason behind this could be the morphology of the two samples (see Table 2). Moreover, the surface area (*S_a_*) of the TiO_2_:Nb crystallites compared to the TiO_2_:V, as determined from XRD (Table 1) is higher, which can contribute to the higher photocatalytic performance of the niobium-doped TiO_2_ thin film. Previously, the authors have also observed that doping with V leads to slightly smaller and better packed spherical particles compared to Nb-doping [28]. A less porous surface can lead to limited pollutant adsorption and degradation, which can explain the smaller photocatalytic efficiency in MB degradation for the TiO_2_:V thin film. Another factor could be due to the imperfect doping mechanism of V, as previously described in [28]. Through XPS analysis, it was shown that the process of incorporating V into the TiO_2_ matrix is less successful compared to Nb (as only 0.8% at. V was found on the film surface, compared to the intended 1.2% at. [28]).

It is well-known that MB bleaches partially under UV–Vis irradiation [71]; therefore, 15 mL of MB solution were irradiated in the same test conditions, but without the addition of the TiO_2_-based photocatalysts. The photodegradation efficiency due to bleaching is approximately 6% after 8 h irradiation, which is quite low. This confirms the additional photocatalytic effect of the doped and undoped TiO_2_ thin films (Figure 15a).

The reaction rate constant, *k*, was calculated as the slope of the linearization of ln(*C*/*C*_0_) versus time, shown in Figure 15b. The reaction rate constants for the three photocatalytic reactions, as well as the photolysis, are given in Table 5.

As can be seen from Figure 15b and Table 5, the Langmuir–Hinshelwood model fits the photodegradation process of MB well when using photocatalysts with high values of R^2^. The reaction rate is the highest when using the TiO_2_:Nb thin film, as it is nearly double that of TiO_2_. However, the overall reaction rate constants are quite low and suggest that the photocatalytic films have limited applicability in large-scale removal of dyes from water. The most promising thin film, TiO_2_:Nb, could become a stronger contender for this application given a higher porosity or more free electrons (slightly higher doping amount).

**Figure 16:**
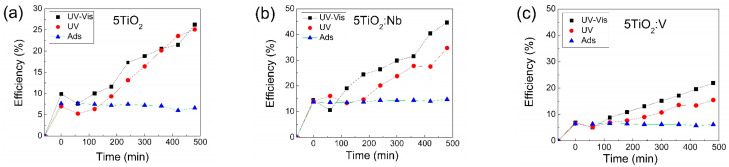
Photocatalytic efficiency in methylene blue solution for the (**a**) 5TiO_2_, (**b**) 5TiO_2_:Nb and (**c**) 5TiO_2_:V thin films deposited on microscopic glass, under UV–Vis, UV and no irradiation (adsorption).

To check the Vis-activation of the samples, photocatalytic tests were also performed under UV irradiation, with no Vis input. It can be seen from Figure 16a that there is no significant difference between the photocatalytic efficiency of pure TiO_2_ under UV–Vis and UV irradiation, respectively. This is due to the fact that TiO_2_ is only activated by the UV radiation and Vis radiation does not have an impact. The slightly higher photocatalytic efficiency under UV–Vis irradiation can be attributed to the photocatalyst sensitization by the methylene blue dye.

In Figure 16b,c, the effect of Nb and V doping can be seen. The photocatalytic efficiency is higher for both TiO_2_:Nb and TiO_2_:V under UV–Vis irradiation compared to UV irradiation. This can be linked to their decreased *E*_og_ compared to pure TiO_2_, allowing for activation under a wider spectral range. Initial adsorption of the pollutant after the first hour of testing (when no radiation is used) plays a significant role as the sample with the highest adsorption (TiO_2_:Nb) is also the one with the highest overall photocatalytic efficiency. However, adsorption can be seen in all three cases (TiO_2_, TiO_2_:Nb and TiO_2_:V) to contribute only in small amount to the overall removal of the MB dye from the solution (Figure 16). The removal efficiency when no radiation is used is relatively constant after the first hour indicating that an equilibrium was reached between the two competing processes namely adsorption and desorption of the MB molecules to and from the photocatalyst surface.

In order to better evaluate the photocatalytic performance of the reported thin films, a comparison is made with some of the most recent reports regarding TiO_2_ doped with Nb or V (Table 6). 

As can be seen, the major focus has been on obtaining doped TiO_2_ powders through chemical methods (e.g., sol-gel, hydrothermal). While powder photocatalysts are more efficient in pollutant removal, due to their larger specific surface, they are much more difficult to separate from water and, therefore, to reuse. For the Nb-doped titania, very good removal efficiencies for 2-propanol have been reported under UV irradiation, but this is not maintained under sunlight-type radiation [72]. This suggests that Vis-activation did not take place in this case. Other reports also give very good degradation efficiency for dyes such as methylene blue (~60% after 45 min) [73] or rhodamine B (100% after 90 min) [74] under UV irradiation, but the amount of photocatalyst used is also significant (10 mg/mL or 50 mg/mL), which cannot compare to the photocatalyst amount used in this work. An impressive 98.5% MB removal was also reported [75] when using Nb and N co-doped TiO_2_ under natural sunlight, after 105 min. This is expected given the high irradiance of the radiation as well as the high photocatalyst load and MB initial concentration (25 ppm).

Regarding the TiO_2_:V reports from the last 5 years, the same trend of using mostly powders can be observed. A report of magnetron-sputtered thin films on Si wafers confirmed that V-doping can extend the activation range from the UV to the Vis as MB removal increased from 75% to 90% [76]. In this case, the irradiance of the light sources was 720 and 1220 W/m^2^, respectively, which is significantly higher than that used in the frame of this work. A higher light intensity can lead to the formation of more reactive oxidative species. Other reports on TiO_2_:V focus on powder or nanoparticle composites [77,78] where the active surface is larger than in the case of thin films, as previously discussed. The addition of carbon derivatives such as g-C_3_N_4_ can prove beneficial as it can both reduce the bandgap energy (being a narrow band semiconductor) and act as a charge scavenger, prolonging the lifetime of electrons and leading to improved photocatalytic efficiency [79].

In conclusion, while the materials reported in this paper are not as efficient in the removal of methylene blue from water under low-intensity radiation, they could become more attractive and more competitive if (a) the overall charge injection in the TiO_2_ matrix by doping can be improved (e.g., by changing the doping amount), (b) the photocatalyst “load” is increased (e.g., by increasing the photocatalyst surface either through increasing porosity and/or increasing the substrate surface, possibly depositing the films on photoreactor walls), or (c) the irradiance of the light source is increased (e.g., by moving towards simulated or natural sunlight).

## 4. Conclusions

Sol-gel multilayered Niobium (Vanadium)-doped TiO_2_ films have been studied for their applicability in gas detection and in photocatalytic processes. Structural, optical, and electrical studies were performed to verify that their properties are suitable for CO detection and photocatalytic methylene blue degradation. 

In the sol-gel nc-TiO_2_Nb(V) films, the crystallites grew in anatase phase with grain sizes in the range of (13–19) nm, as revealed by XRD. 

SE studies showed reduced optical bandgap and enhanced porosity for Nb(V)-doped TiO_2_ films compared to pure TiO_2_, a prerequisite for the applications mentioned above. 

The electrical characterization of Si-TiO_2_:Nb(V) MIS structures revealed that the introduced Nb and V donors are compensated by acceptor levels generated in the films during the sol-gel technological processes. These levels may contribute to the observed bandgap narrowing of the TiO_2_ energy gap. It is shown that in the studied Si-TiO_2_:Nb(V) MIS structures, the charge carriers transport is mainly by inter-trap tunneling via deep levels in the TiO_2_ bandgap. 

The tested sensors with Nb(V)-doped sol-gel TiO_2_ multilayers showed improved CO-sensing properties compared to the sensor based on pure TiO_2_. Although the TiO_2_:Nb-based sensor performed better at lower concentration (250 ppm CO in air), the TiO_2_:V-based sensor showed better overall CO-sensing results (up to the studied 2000 ppm CO in air), confirming the CO detection capability of the Vanadium-doped sol-gel TiO_2_ films. These results open a new perspective for V-doped sol-gel TiO_2_ as a CO sensor for future low-budget chemiresistor development.

The testing of sol-gel doped and undoped TiO_2_ films as photocatalysts for the removal of MB from water under UV–Vis irradiation revealed that the photocatalytic efficiency of a Nb-doped TiO_2_ thin film was nearly twice as high as that of pure or V-doped TiO_2_, making the TiO_2_:Nb thin films the most promising for up-scaling.

## Figures and Tables

**Table 1:** Characteristic parameters, as the lattice constants *a* = *b* and *c*, average crystallites size (*D*), and parameters of the anatase structural units, as density (*ρ*) and volume of unit cell and specific surface area of grains (*S_a_*), in the sol-gel TiO_2_, TiO_2_:Nb and TiO_2_:V layers.

Samples	2θ_Pmax_ (Degree)	β _FWHM_ (Degree)	D (nm)	Miller Indices (hkl)	d-Spacing (Å)	Lattice Parameters		Unit Cell Volume (Å^3^)	*ρ* (g/cm^3^)	*S_a_* (cm^2^/g)
						*a* = *b* (Å)	*c* (Å)			
TiO_2_	25.42	0.4621	18.40	(101)	3.5010	3.76974	9.4419	134.178	395.43	0.827 × 10^4^
	38.09	0.49514	17.71	(004)	2.3605		9.4419			
	48.24	0.568	16.01	(200)	1.8849	3.76974				
	54.246	0.62733	14.86	(105)	1.6903					
	55.293	0.5536	16.91	(211)	1.6610					
	62.9	0.686	14.17	(204)	1.4763					
TiO_2_:V	25.419	0.587	14.50	(101)	3.501	3.7700	9.43715	134.129	395.58	1.046 × 10^4^
	38.11	0.6199	14,17	(004)	2.3593		9.43715			
	48.236	0.66548	13.67	(200)	1.8850	3.7700				
	54.25	0.63701	14.63	(105)	1.6894					
	55.293	0.65198	14.36	(211)	1.660					
	62.914	0.749	12.98	(204)	1.4743					
TiO_2_:Nb	25.42	0.61274	13.89	(101)	3.501	3.7683	9.46106	134.348	394.93	1.094 × 10^4^
	38.01	0.63792	13.76	(004)	2.3653		9.46106			
	48.236	0.74017	12.29	(200)	1.8841	3.7683				
	54.20	0.7083	13.15	(105)	1.6908					
	55.12	0.72019	12.99	(211)	1.6648					
	62.91	0.7186	13.53	(204)	1.4778					
Anatase TiO_2_ (card JCPDS 21-1272)	25.30			(101)	3.520	3.7845	9.5143	136.268	389.45	
	37.62			(004)	2.378					
	48.03			(200)	1.892					
	53.9			(105)	1.699					
	55.12			(211)	1.6665					
	62.75			(204)	1.4808					

**Table 2:** Thickness (*d_film_*, *d_rough_*), porosity (*P*) and optical conductivity (*σ*) of TiO_2_ and TiO_2_:Nb(V) multilayers on c-Si substrates.

Sample	*d_film_* (nm)	*d_rough_* (nm)	*P* (%)	*E_og_* (eV)	*σ* at 370 nm (Ωcm)^−1^
5TiO_2_	174.4	1.4	2.81	3.31	25.65
10TiO_2_	386.2	0.5	1.90	3.28	11.7
5TiO_2_:Nb	170.1	1.9	11.73	3.15	36.05
10TiO_2_:Nb	340.2	0.7	8.82	2.7	59.29
5TiO_2_:V	268.9	0.9	18.21	2.94	44.61
10TiO_2_:V	534.2	0.3	15.94	3.12	30.55

**Table 3:** Specific resistivity *ρ*, effective electron mobility *μ_ef_*, diffused electron concentration *n_e_*, sheet energy density of deep levels *N_ss_*, and bulk energy density of traps *N_tb_*, of the 5TiO_2_:Nb(V) and 10TiO_2_:Nb(V) films.

Quantities	Parameters	5TiO_2_:Nb	10TiO_2_:Nb	5TiO_2_:V	10TiO_2_:V
Specific resistivity	*ρ* (Ωcm)	5.6 × 10^4^	6.3 × 10^4^	4.8 × 10^4^	6.1 × 10^4^
Effective electron mobility	*μ_ef_* (cm^2^V^−1^s^−1^)	5.1 × 10^−3^	1.7 × 10^−3^	3.3 × 10^−3^	3.9 × 10^−3^
Diffused electron concentration	*n_e_*	2.2 × 10^16^	5.7 × 10^16^	3.9 × 10^16^	2.6 × 10^16^
Dopant concentration	*N_d_* > *n_e_* (cm^−3^)	≥2.2 × 10^16^	≥5.7 × 10^16^	≥3.9 × 10^16^	≥2.6 × 10^16^
Sheet energy density of traps	*N_ss_* (cm^−2^eV^−1^)	1.0 × 10^12^ (at −3 V)	1.3 × 10^12^ (at −2.5 V)	4.1 × 10^11^ (at −15 V)	1.8 × 10^11^ (at −10 V)
Bulk energy density of traps	*N_tb_* (cm^−3^eV^−1^)	5.9 × 10^16^ (at −3 V)	3.7 × 10^16^ (at −2.5 V)	1.5 × 10^16^ (at −15 V)	3.4 × 10^15^ (at −10 V)
Acceptor type deep levels	*N_t_* (cm^−3^)	6.8 × 10^19^	3.6 × 10^19^	4.4 × 10^19^	2.1 × 10^19^

**Table 4:** Effects of CO exposure, taking into account the gas concentration and exposure time, adapted from reference [59].

CO Concentration (ppm)	Exposure Duration (h)	CO Exposure Effects	Source [60,61,62]
0–4	8	Good air quality.	EPA
35	8	Recommended 8 h max. workplace exposure.	US NIOSH
50	8	Recommended 8 h max. workplace exposure.	US OSHA PEL
400	1–3	Healthy adults will suffer from headaches, dizziness, and nausea after 1–2 h. Life-threating after 3 h.	CDC/NIOSH
800	<2	Healthy adults will suffer from headaches, dizziness, and nausea after 45 min. Unconsciousness/death in less than 2 h.	CDC/NIOSH
3000	<30 min	Death in less than 30 min	CDC/NIOSH

**Table 5:** Reaction rate constants and *R*^2^ from the fitting of ln(*C*/*C*_0_) versus time, for the photodegradation of MB solution (10 ppm) with addition of TiO_2_, TiO_2_:Nb, TiO_2_:V or no photocatalyst.

TiO_2_		TiO_2_:Nb		TiO_2_:V		MB	
*k* (min^−1^)	*R* ^2^	*k* (min^−1^)	*R* ^2^	*k* (min^−1^)	*R* ^2^	*k* (min^−1^)	*R* ^2^
6.48 × 10^−4^	0.9899	12.2 × 10^−4^	0.9812	5.37 × 10^−4^	0.9853	1.71 × 10^−4^	0.8145

**Table 6:** Literature review of TiO_2_:Nb and TiO_2_:V photocatalysts.

Crt. No.	Photocatalyst Type	Synthesis Method	Doping Amount	Photocatalytic Experiments Conditions (Reported as Optimal)	Radiation Type	Photodegradation Efficiency	Ref.
1.	TiO_2_:Nb powders	Sol-gel	0.5 at. %	Pollutant: 2-propanol, 700 ppmv Photocatalyst load: 1 mg/cm^−2^ coated on the photoreactor walls	UV and sunlight-type (SL)	33% under UV irradiation <5% under SL irradiation	[72]
2.	TiO_2_:Nb powders	Hydrothermal	0.27 at. %	Pollutant: methylene blue (10 ppm) Photocatalyst load: 10 mg/30 mL	UV	~60% after 45 min *K* = 0.02055	[73]
3.	TiO_2_/Nb_2_O_5_ powders	Microwave-assisted hydrothermal	Not specified	Pollutant: rhodamine B (10^−5^ M) Photocatalyst load: 50 mg/50 mL	UV	100% after 90 min *K* = 0.4195 min^−1^	[74]
4.	Nb-N-TiO_2_ powders	Sol-gel	5 wt. %	Pollutant: methylene blue, 25 ppm Photocatalyst load: 45 mg/70 mL pH: 11	Sunlight	98.5% after 105 min	[75]
5.	TiO_2_/V_2_O_5_ thin films on Si	Magnetron sputtering, anodization, heat treatment	1 wt. %	Pollutant: methylene blue (5 ppm) Photocatalyst load: not specified	UV Vis UV–Vis	75% after 270 min 40% after 270 min 90% after 270 min	[76]
6.	V-TiO_2_ powders	Microwave assisted sol-gel	1.9 wt. %	Pollutant: methylene blue (10 ppm) Photocatalyst load: 0.1 g/50 mL	UV–Vis	98.5% after 60 min	[77]
7.	TiO_2_:V NP	Sol-gel hydrolysis	V:Ti = 0.3	Pollutant: methylene blue (<10 ppm) Photocatalyst load: 5 mg/100 mL	Vis	60% after 210 min	[78]
8.	TiO_2_/V_2_O_5_/g-C_3_N_4_ powders	Hydrothermal	Not specified	Pollutant: methyl orange (0.1 mM) methylene blue (0.1 mM) Photocatalyst load: 10 mg/25 mL	sunlight	94% after 180 min (*k* = 0.0165 min^−1^) 89% after 90 min (*k* = 0.005 min^−1^)	[79]
9.	TiO_2_:Nb thin films TiO_2_:V thin films	Sol-gel	1.2 at. %	Pollutant: methylene blue (10 ppm) Photocatalyst load: 1.5 cm^2^/15 mL	UV UV–Vis	45% (UV–Vis) and 35% (UV) after 8 h for TiO_2_:Nb 22% (UV–Vis) and 15% (UV) after 8 h for TiO_2_:V	This work

## Data Availability

Data are contained within the article.
